# Phototherapy Using a Light-Emitting Fabric (BUBOLight) Device in the Treatment of Newborn Jaundice: Protocol for an Interventional Feasibility and Safety Study

**DOI:** 10.2196/24808

**Published:** 2021-05-25

**Authors:** Fabienne Lecomte, Elise Thecua, Laurine Ziane, Pascal Deleporte, Alain Duhamel, Clémence Vamour, Serge Mordon, Thameur Rakza

**Affiliations:** 1 U1189 - Assisted Laser Therapy and Immunotherapy for Oncology Univ- Lille, Inserm, CHU Lille F-59000 Lille France; 2 EA 2694 - Santé publique: épidémiologie et qualité des soins Univ- Lille, CHU Lille F-59000 Lille France; 3 Jeanne de Flandre Hospital, Department of Obstetrics CHU Lille F-59000 Lille France; 4 EA4489, Perinatal Growth and Health Jeanne de Flandre Hospital, Department of Obstetrics Univ- Lille, CHU Lille F-59000 Lille France

**Keywords:** jaundice, light emitting fabrics, light, neonate, newborn jaundice, perinatal, phototherapy

## Abstract

**Background:**

Neonatal jaundice is a common condition occurring in 60%-80% of all healthy-term and late-preterm neonates. In the majority of cases, neonatal jaundice resolves spontaneously and causes no harm; however, in some neonates, signiﬁcant hyperbilirubinemia can develop and lead to kernicterus jaundice, a serious neurological disease. Phototherapy (PT) is the preferred treatment for jaundice; however, to be effective, PT devices need to have a broad light emission surface to generate no or little heat and to provide an optimal wavelength and light intensity (420-490 nm and ≥30 µW/cm²/nm, respectively).

**Objective:**

This study aimed to investigate the feasibility, safety, and level of satisfaction of parents and health care teams with the BUBOlight device, an innovative alternative to conventional hospital PT, in which luminous textiles have been incorporated in a sleeping bag.

**Methods:**

This interventional, exploratory, simple group, nonrandomized, single-center trial will be conducted at Lille Hospital. In total, 10-15 neonates and their parents will be included to obtain evaluable data from 10 parent-neonate pairs. Neonates weighing more than 2500 g at birth and born with ≥37 weeks of amenorrhea that required PT in accordance with the guidelines of the National Institute For Health and Clinical Excellence will receive one 4-hour session of illumination. Total serum bilirubin and transcutaneous bilirubin levels were obtained at the start and 2 hours after the end of PT. Cutaneous and rectal temperatures, heart rate, and oxygen saturation will be measured at the beginning and during PT. The number of subjects is therefore not calculated on the basis of statistical assumptions. We aim to obtain a minimum proportion of 90% (ie, 9 of 10) of the neonates included, who have been able to undergo 4-hour PT without unacceptable and unexpected toxicities. We will calculate the mean, median, quartiles, minimum and maximum values of the quantitative parameters, and the frequency of the qualitative parameters. The rate of patients with no unacceptable and unexpected toxicities (ie, the primary endpoint) will be calculated.

**Results:**

The first patient is expected to be enrolled at the end of 2020, and clinical investigations are intended for up to June 2021. The final results of this study are expected to be available at the end of 2021.

**Conclusions:**

Our findings will provide insights into the safety and feasibility of a new PT device based on light-emitting fabrics for the treatment of newborn jaundice. This new system, if proven effective, will improve the humanization of neonatal care and help avoid mother-child separation.

**Trial Registration:**

ClinicalTrials.gov NCT04365998; https://clinicaltrials.gov/ct2/show/NCT04365998

**International Registered Report Identifier (IRRID):**

PRR1-10.2196/24808

## Introduction

Neonatal jaundice or hyperbilirubinemia is very common in neonatology. Neonatal livers are generally immature, and this condition leads to poor metabolism of bilirubin. Bilirubin is not sufficiently degraded and accumulates excessively in the blood. This clinically manifests as yellowing of the skin and mucous membranes. Based on etiology, jaundice can be divided into physiological and pathological jaundice [1,2].

The evolution of pathological jaundice is often favorable, but it can lead to complications such as acute or even chronic encephalopathy known as kernicterus [3].

Hyperbilirubinemia from any cause among healthy infants is considered to be of concern if the bilirubin level is >18 mg/dL (>308 μmol/L) in infants who are 49-72 hours old [4] and requires prompt management. Furthermore, jaundice is the primary cause of rehospitalization in the first 15 days of life, which makes it an important issue for health professionals.

Screening and diagnosis of jaundice is routinely performed for all neonates. It is based on a daily visual clinical assessment, which must be combined with a transcutaneous bilirubin measurement or total serum bilirubin measurement as only 50% of neonates with a total serum bilirubin concentration of >128 μmol/L visually appear to have jaundice, especially dark-skinned neonates [5].

Bilirubin is a yellow pigment that preferentially absorbs blue, violet, or green light (400-490 nm) [6]. Phototherapy (PT) is used as first-line treatment for hyperbilirubinemia. The aim of PT is to decrease or prevent an increase in the concentration of circulating bilirubin by using blue light.

Light absorption by bilirubin induces the formation of photoisomers that can be excreted in the urine or bile, thus bypassing hepatic conjugation [7,8].

The indication for PT depends on the total bilirubin levels in blood and the presence or absence of conditions that increase the risk of bilirubin neurotoxicity.

Intensive PT, defined by an irradiance of ≥30 µW/cm^2^/nm, is more effective than conventional PT [9], but there is no standardized method for delivering efficient PT. Nonetheless, PT for a short duration (ie, 4 hours) by illuminating the largest body surface bas been recommended [10].

There are several PT devices that contain light-emitting diode–based lights, conventional fluorescent blue lights, or conventional halogen lights with an emission spectrum of 420-490 nm, which can provide effective irradiance to reduce serum bilirubin levels [11], but characteristics such as the distance of the light source from the neonate, the area illuminated, and the irradiance affect the effectiveness of phototherapy [12].

The BUBOLight PT device is composed of a light source and light-emitting fabrics (LEFs) in a sleeping bag. LEFs incorporate optical fibers inside a textile structure. By controlling the density of the fibers and the bending angles, this structure makes it possible to obtain a homogeneous light distribution on a very flexible textile surface.

BUBOLight emits light with a wavelength of 445 nm, an irradiance equal to 3.5 mW/cm², and a useful surface of 756 cm². It thus has the characteristics of an effective PT; that is, a sufficiently exposed surface, a wavelength similar to the maximum absorption peak of bilirubin, a sufficiently high irradiance, bilateral illumination to increase the total treatment surface, and a homogeneous and stable light distribution.

In addition, BUBOLight has been designed not to heat up, thus avoiding the risk of hyperthermia in neonates. When used in the infant's bed, it helps preserve parent-child interactions and does not require the wearing of protective glasses because the light cannot pass through the sleeping bag. Finally, the infant is installed with a special diaper that includes protection for the genitals. The aim of the present protocol is to evaluate the feasibility and the safety of the BUBOLight device as a new PT method.

## Methods

### Trial Design

This interventional, exploratory, simple group, nonrandomized, single-center study will include 10-15 neonates in order to obtain evaluable data from 10 parent-child pairs.

### Setting

The study will be conducted at the department of obstetrics at Lille University Hospital (Lille, France), over a period of 6 months until June 2021. Parent-neonate couples will be included and followed-up in the study until neonatal serum bilirubin levels are measured 2 hours after the end of PT. The participation of parent-child pairs in this study will then be limited to a 6-hour session.

### Device

**Figure 1:**
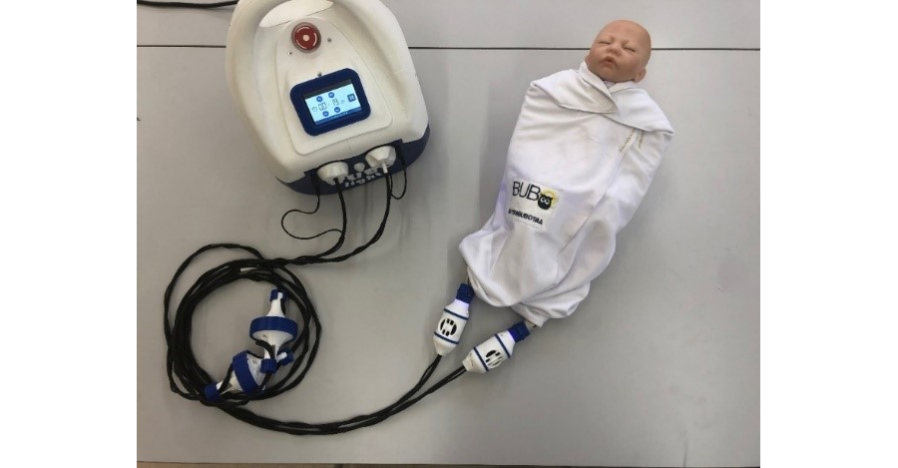
Complete device and light source.

**Figure 2:**
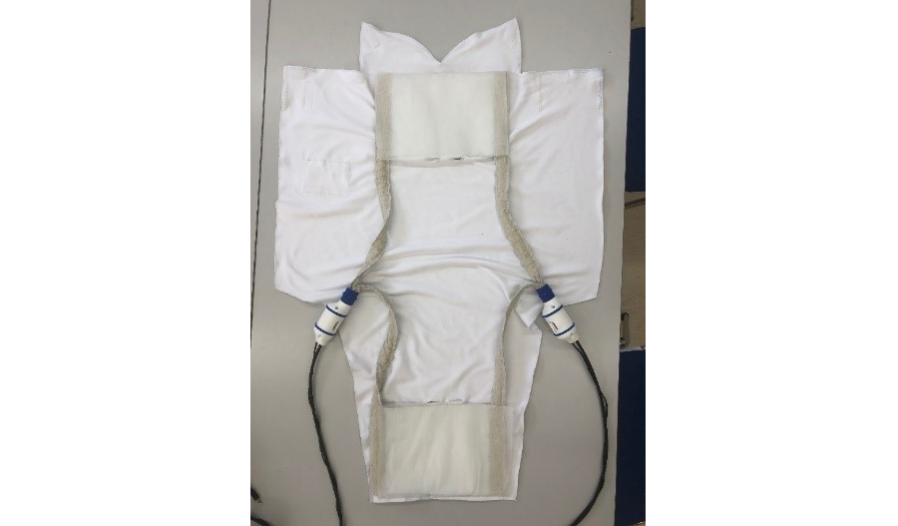
Light-emitting fabrics in the sleeping bag.

The complete BUBOLight device consists of an electrically powered light source that emits blue radiation with a predefined wavelength of 445 nm connected to an active textile part (Figure 1). The active textile part of the device consists of sleeping bag system in which are integrated 2 removable LEFS (Figure 2) to diffuse a blue light from a light source. The light source was designed by the ONCO-THAI U1189 (Lille, France) and is composed of 2 light sources with an output power of 4 W. The active textile part (MDB Texinov) has a usable surface area of 756 cm² and can deliver an irradiance of 3.5 mW/cm² (with a variation of 20%) in blue light.

### Participants

To be eligible for the study, neonates must meet all the inclusion criteria described in Textbox 1. Neonates must not meet any of the exclusion criteria.

**Inclusion and exclusion criteria.**

**Inclusion criteria**

- Infants born at the Lille University Hospital and not out of this hospital
- Gestational age of ≥37 weeks with amenorrhea 
- Presence of jaundice confirmed through transcutaneous bilirubin measurement with a bilirubinometer (model JM-105, Dräger) (Reference graphs related to gestational age–specific thresholds for initiating PT treatment [13])
- Rate of total serum bilirubinemia requiring phototherapy (Reference graphs related to gestational age–specific thresholds for initiating PT treatment [13])
- Absence of rhesus or Kell fetal-maternal incompatibility
- Weight at birth of ≥2.5 kg
- Discerned to be in good health by the investigator after clinical examination and on the basis of medical data (absence of perinatal asphyxia, antibiotic treatment, and respiratory disorders)

**Exclusion criteria**

- Neonate who has already been treated with PT
- Febrile state with a body temperature of >37.5°C
- Total bilirubinemia rate or excess of 100 µmol/L as an indication for PT
- Neonates with jaundice due to hemolysis or functional or anatomical obstruction
- Weight loss of >10% of the birth weight
- Neonates requiring treatment other than PT
- Neonates with congenital erythropoietic porphyria or a family history of porphyria.
- Presence of ≥2 of the following risk factors:
  Gestational age of <38 weeks of amenorrhea 
  Icterus of the first 24 hours
  ABO incompatibility
  Positive irregular agglutinin test status of the mother
  History of jaundice treated in siblings
  History of familial hemolysis
  Serosanguineous bump, bruise, cephalohematoma
  Ineffective breastfeeding
  Weight loss of ≥8%
- Parents who are noncompliant with the study design

### Study Objectives and Outcomes

The primary objective of the study is to evaluate the safety of the BUBOLight PT device as an alternative to conventional tunnel PT under the usual conditions for the management of jaundice in neonates.

Safety will be based on the proportion of neonates who received complete and effective 4-hour PT with the BUBOlight device and did not experience unacceptable and unexpected toxicities (target set at least 90% of neonates).

The key secondary objectives are the individually frequency of each adverse effect, monitoring of serum bilirubin and transcutaneous levels under phototherapy, and the perceptions of parents and health team with the use of the device (comfort, heat, humidity, ease of breastfeeding, proximity, and possibility of contact with the baby) and causes of PT discontinuation. Table 1 summarizes the study objectives and outcomes.

**Table 1:** Study objectives and outcomes.

Outcomes		Inclusion: initiation of phototherapy (hour 0)	1 hour after the beginning of phototherapy	2 hours after the beginning of phototherapy	4-hour effective phototherapy	2 hours after the end of phototherapy
**Primary outcomes: safety**						
	Monitoring of hyperthermia (body temperature of ≥38°C) or hypothermia (body temperature of ≤36°C)	✓	✓	✓	✓	
	Oxygen saturation of <90% for >15 seconds	✓	✓	✓	✓	
	Heart rate of >160 beats/minute during inactivity or <80 beats/minute for >15 seconds	✓	✓	✓	✓	
	Allergic contact reaction of grade ≥3 (Ring and Messmer classification [14])	✓	✓	✓	✓	
**Secondary outcomes**						
	Description of the number of neonates presenting each adverse event individually (skin lesion, dehydration, diarrhea, etc)	✓	✓	✓	✓	
	Monitoring of serum bilirubin levels	✓				✓
	Monitoring of transcutaneous bilirubin levels	✓				✓
	Evolution of the EDIN^a^ score: neonatal pain and discomfort scale	✓			✓	
	Number of feedings, number of diaper changes and the causes of cessation of phototherapy			✓	✓	
	Perceptions of the parents with the use of the device and interaction with their child					✓

^a^EDIN: Échelle de Douleur et d'Inconfort du Nouveau-né.

### Sample Size

This is a pilot feasibility study. The number of subjects is therefore not calculated on the basis of statistical assumptions. We propose to include 10-15 neonates and their parents in order to obtain evaluable data from 10 parent-neonate pairs. The feasibility objective is to obtain a minimum proportion of 90% (ie, 9 of 10) of the neonates included in this study, having undergone PT without unacceptable and unexpected toxicities.

### Allocation and Randomization

There will be no randomization in this study; all neonates will receive PT with the BUBOLight device.

### Implementation and Blinding

The study will not involve blinding as it is an uncontrolled clinical trial with a single group of patients receiving the same treatment. Data will also be analyzed without blinding.

### Intervention

Neonates who have not been discharged from the hospital and who require PT for jaundice will be included in the study after verification of the inclusion and exclusion criteria.

**Figure 3:**
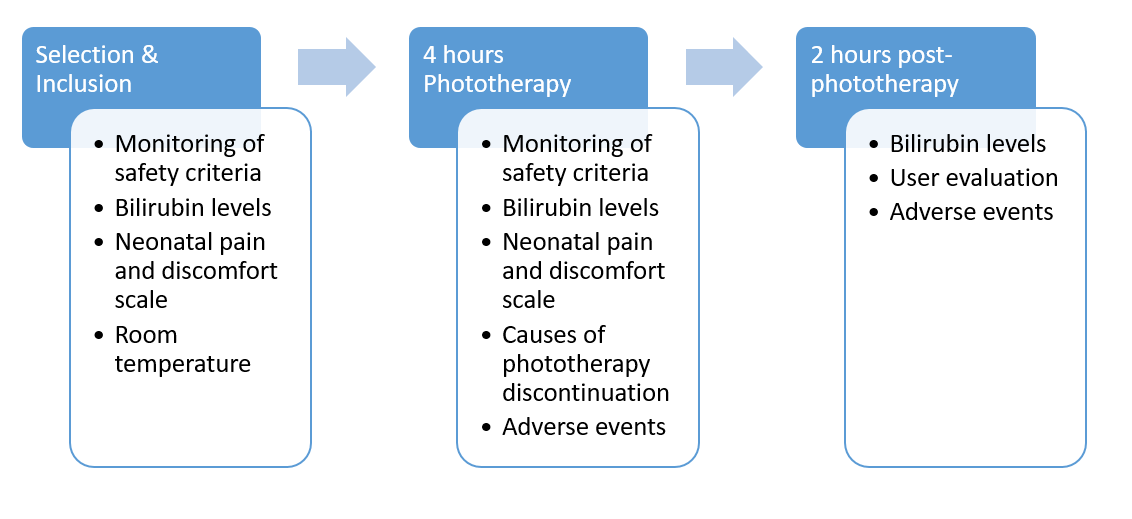
Study design and data collection.

As shown in Figure 3, parent-neonate pairs who meet all of the inclusion criteria and none of the exclusion criteria are invited to participate in the study, which will involve a single visit.

The study will include an effective 4-hour PT session (which may be discontinued for a few minutes to change diapers if necessary) followed by a 2-hour rest period in accordance with the recommendations of the French National Reference Center for Perinatal Hemobiology. Serum bilirubin levels will be determined upon initiation of PT (H0) and 2 hours after the effective end of PT; that is, at approximately H0+6 hours, in order to check for a reduction in bilirubin levels. Transcutaneous measurement with a bilirubinometer (Dräger Jaundice Meter JM-105) will be also performed upon initiation of PT and 2 hours after the effective end of PT.

Heart rate and oxygen saturation will be continuously recorded using a monitor, which is usually used during PT. Alarms will be set to detect desaturation and bradycardia episodes lasting >15 seconds. The investigator will check the scope at the end of the monitoring period for false episodes related to an artefact.

In addition, axillary and cutaneous temperatures will be regularly monitored in order to prevent any risk of dehydration and hyperthermia. Room temperature will also be measured.

The neonatal pain and discomfort scale (Échelle de Douleur et d'Inconfort du Nouveau-né [EDIN] score) and the opinion of parents and health professionals on the use of the device will also be sought.

### Variables and Data Collection

The collected medical data will consist of demographic and medical data (sex, weight, height, day of birth, and gestational age), risk factors for severe hyperbilirubinemia, and the blood type of the mother and the child.

To achieve the primary and secondary endpoints, we will measure serum and transcutaneous bilirubin levels.

Temperature, oxygen saturation, and heart rate data will be monitored throughout the PT session. EDIN scores of 0-15 will be considered for monitoring neonatal pain and discomfort, and data regarding PT discontinuation will be collected (including the number of changes, feedings, and cares).

Parents and medical staff will be asked to complete feedback questionnaires at the end of the observation period. Data will be collected on device usability, assessment of noise, and the design of the device.

### Data Management

This study complies with methodology MR-001 of the Commission nationale de l'informatique et des libertés for the treatment of personal data, which is a simplified declaration of data from medical research to the French National Data Protection Authority. The only persons authorized to access data and modify the files generated in this study are those who are directly involved in the study. These participants will have access to data and be able to modify them at any moment in consultation with one of the referring investigators of the study. The sponsor affirms the patient’s right to protection against invasion of privacy.

The data will be collected through a case report form and be saved in an electronic file (database). All participants will receive a trial identifier, and only the investigator knows the personal details. The sponsor’s monitor will plan several monitoring visits, after initial enrollment at the study site and periodically to assess data quality and study integrity. The sponsor’s monitor will review the study records and directly compare them with the source documents, discuss the conduct of the study with the investigator, and verify that the facilities remain acceptable. The trial will be monitored in accordance with the monitoring plan. A planning meeting with the principal investigator will hence be held before the start of the trial. During the trial, several checkpoints are defined, including the presence of signed informed consent forms obtained by the investigator, adherence to the inclusion and exclusion criteria, reporting of any adverse events, and the monitoring of all steps of patient follow-up. At the end of the trial and once the final analysis is completed and validated, all the files are sealed and archived in accordance with specific procedures at a secure location at the clinical research department of the sponsor.

The trial support unit will coordinate the data management. The database is stored and secured on the network of Lille University Hospital. Before the closeout of the database, data monitoring will be performed using XLSTAT software (Addinsoft Inc) in accordance with consistency guidelines set with the project manager (eg, missing data, outliers, and inconsistency among several variables). The data will be analyzed at the OncoTHAI Laser Assisted Therapies and Immunotherapies for Oncology unit (U1189, Inserm, CHU Lille). Only the investigator participating in the study or a collaborator designated by the physician and participating in the study may modify the data. The study data will be archived for a minimum period of 15 years from the end of the study or its early termination without bias toward the laws and regulations in force.

### Statistical Analysis

Security and acceptability will be assessed using descriptive data. All data will be described individually and summarized using the following statistical parameters:

*Primary objective:* the mean, median, quartiles, minimum and maximum values of the quantitative parameters, and the frequency of the qualitative parameters will be calculated. The rate of patients with no unacceptable and unexpected toxicities (primary endpoint) will be calculated.

*Secondary objectives:* bilirubin levels and EDIN score data for neonatal pain and discomfort will be expressed as means, medians, quartiles, and minimum and maximum values at each measurement time.

Changes in serum and transcutaneous bilirubin levels among PT initiation (H0), H0+4 hours (only transcutaneous), and 2 hours after the end of PT, and changes in the EDIN score between PT initiation (H0) and after 4 hours of actual treatment will also be described. Furthermore, we will calculate the mean, median, quartiles, minimum and maximum values of the quantitative parameters (number of feedings, diaper changes, and care), and the frequency of the qualitative parameters (perceptions of the parents and the health team regarding the use of the device and interaction with their infant).

### Ethical Consideration

The trial will be conducted in accordance with tenets of the Declaration of Helsinki and the guidelines of the International Council for Harmonization and article L1121-4 of the Public health code. The study protocol has been submitted for review and approval by the French Ethics Committee (protocol# 20/025-1) and the French National Agency for the Safety of Medicines and Health Products (protocol# 2019-A01417-50). The trial was registered at ClinicalTrials.gov (protocol# NCT04365998). The investigator must ensure that the parents of the subjects are informed clearly and thoroughly about the purpose, potential risks, and other critical issues related to the trial in which they volunteer to participate. Written informed consent must be freely obtained from each parent of the subjects prior to their participation in the trial, including informed consent for any screening procedure conducted to establish subject eligibility for the study. The rights, safety, and well-being of the parent-neonate pair are the most important considerations and should prevail over the interests of science and society.

## Results

The first parent-neonate pair will be enrolled at the end of 2020. All data collected will provide a basis to analyze the safety and effectiveness of the device. The last subject is expected to be enrolled by June 2021. Analysis of the data and results are expected to be completed at the end of 2021.

## Discussion

PT using light emitting diode light tunnels will inevitably lead to a physical and psychological distance, which will interfere with mother-neonate bonding, potentially cause problems with breastfeeding, and increase the exposure to infections. The development of new PT systems that are as effective as conventional PT is therefore necessary.

BUBOLight has been designed to incorporate LEFs to deliver PT directly on the neonate’s skin in his/her sleeping bag, thus allowing the mother to change and breastfeed the neonate without interrupting treatment, in the hospital environment. In case we obtain positive results, we hope to initiate a comparative study of BUBOLight versus conventional PT devices in order to use BUBOLight intermittently or exclusively in outpatient follow-up programs.

## Abbreviations

EDIN: Échelle de Douleur et d'Inconfort du Nouveau-né
LEF: light-emitting fabric
PT: phototherapy
